# AMPK-ERK/CARM1 Signaling Pathways Affect Autophagy of Hepatic Cells in Samples of Liver Cancer Patients

**DOI:** 10.3389/fonc.2019.01247

**Published:** 2019-11-14

**Authors:** Qiu-Fang Qin, Xiao-Jun Li, Yu-Sang Li, Wei Kevin Zhang, Gui-Hua Tian, Hong-Cai Shang, He-Bin Tang

**Affiliations:** ^1^Lab of Hepatopharmacology and Ethnopharmacology, School of Pharmaceutical Sciences, South-Central University for Nationalities, Wuhan, China; ^2^Key Laboratory of Chinese Internal Medicine of MOE, Beijing Dongzhimen Hospital, Beijing University of Chinese Medicine, Beijing, China

**Keywords:** AMPK, autophagy, 4-HNE, liver cancer, poorly differentiated

## Abstract

Liver cancer is one of the most common malignant tumors, with the death rate ranking fourth among all types of cancer. Over the past few decades, several studies have reported that liver tumorigenesis is associated with dysfunction in autophagy. However, the detailed mechanism remains unclear. In this paper, we used tissue micro-array (TMA) of liver cancer to detect proteins associated with the regulation of autophagic signaling in non-cancerous and cancerous regions by immunohistochemical staining. Those proteins contained 4-HNE, p-AMPK, Erk1/2, p-Erk1/2, CARM1, TFEB, LAMP1, and p62. According to the degrees of tumor differentiation in patients (well differentiated group vs. moderately and poorly differentiated group), we analyzed each protein's expression in the ratio of the “cancerous region/non-cancerous region” in two groups. Current data showed that there were AMPK-ERK/CARM1 autophagic signaling pathways during the formation of liver cancer. The above-mentioned changes in signals indicated an upregulation of autophagy in cancerous regions, which means overactivated autophagy plays an important role in liver cancer.

## Introduction

According to the statistics of the World Health Organization (WHO)^1^, cancer is a main factor to cause the death globally, estimating 9.6 million deaths in 2018. As the most common malignant tumors, liver cancer shows a death rate ranking fourth (782,000 deaths) among all types of cancer. Recently, more and more researches have focused on the involvement of autophagy in liver cancer. However, the detailed mechanism still requires further clarification.

Autophagy is an evolutionarily conserved pathway via lysosomes for the degradation of organelles and cytoplasmic proteins, and plays important roles in many diseases, such as clearance of dysfunctional mitochondria and protein aggregates (1), and damaged proteins and organelles (2). Particularly, changes in autophagic activity during the process of oxidative stress can regulate the effects of hydroxynonenal (HNE) on cellular bioenergetics and an impact on cell death was then occurred (3). The downstream effects of 4-HNE, a typical lipid aldehyde, are both multifactorial and cell-specific. Moreover, 4-HNE is also a major indicator of inflammation and oxidative stress, and is considered to initiate cellular damage and promote cellular survival, simultaneously (4). The inhibitory effect of 4-HNE on AMP-activated protein kinase (AMPK) has been reported previously (5).

AMPK is a crucial energy sensor and regulates cellular metabolism to maintain energy homeostasis, and can further promote the autophagy (6). The activity of autophagy is evidenced by elevating AMPK phosphorylation. It has been known to activate autophagy through inhibiting the activity of rapamycin complex 1 (mTORC1), a mammalian target, by directly phosphorylating its receptor and activating phosphorylation of Unc-51-like autophagy activating kinase 1 (ULK1) at early times of starvation (7). Wang et al. (8) has demonstrated that AMPK is an upstream regulator of extracellular signal-regulated kinase (ERK) in autophagy. The ERK (also known as Erk1/2) signaling pathway is indispensable in controlling diverse cellular processes (9). Erk1/2 also participates in the regulation of autophagy. It influences autophagosome formation and adjusts gene expression for cellular turnover as well as autophagy protein synthesis (10), and promote the hepatic autophagy both *in vivo* and *in vitro* (11, 12).

In another downstream of AMPK, co-activated factor-related arginine methyltransferase 1 (CARM1) Shin et al. (13) found that CARM1 expression in various tested cell lines could be increased by the starvation of amino acid or the treatment of rapamycin. An AMPK-SKP2-CARM1 signaling axis was also identified for the first time, in which CARM1 expression in the nucleus could be increased by the nutrient starvation-induced AMPK. Moreover, CARM1-dependent histone arginine methylation, functioning as a key nuclear event of autophagy for epigenetic and transcriptional regulation was further confirmed. Other autophagic proteins, such as the transcription factor EB (TFEB), lysosome-associated membrane protein 1 (LAMP1), and p62/SQSTM (sequestosome) was also detected in our present study. We hypothesize that the AMPK-ERK/CARM1 signaling pathways play important roles in the progression of liver cancer.

## Materials and Methods

### Reagents

The used reagents were summarized as follows: SQSTM1/p62 (Abcam, UK), LAMP1-lysosome marker (Abcam, UK), p44/42 MAPK (Erk1/2; Cell Signaling, US), p-AMPKα1/2 Thr172 (Santa Cruz Biotechnology, US), phospho-p44/42 MAPK (Erk1/2; Thr202/Tyr204; Cell Signaling, US), anti-4 hydroxynonenal antibody (Abcam, UK), rabbit monoclonal to PRMT4/CARM1 (C31G9), rabbit mAb (Cell Signaling, US), goat polyclonal to TFEB-ChIP grade (Abcam, UK), and horseradish peroxidase with a substrate solution of 3,3′-diaminobenzidine tetrahydrochloride (DAB) (Nichirei, Japan).

### Human Samples and Their Tissue Micro-Array (TMA) Analysis

Liver tissue samples of 30 liver cancer patients were obtained from the Key Laboratory of Chinese Internal Medicine of MOE of the Beijing Dongzhimen Hospital of Beijing University of Chinese Medicine. The cancerous region was obtained in carcinoma tissues and reviewed by professional pathologists. The non-cancerous region was obtained in normal tissues over 3 cm apart from the borderline of the cancerous region in the same fixed slice. The collection and follow-up manipulation of all pathological tissue samples were approved by the Committee on the Ethics of Experiments of the South-Central University for Nationalities in China (Permit Number: 2017-SCUEC-MEC-007).

The tissues were extracted using a tissue chip handle (2 μm), then put into a 96-hole paraffin mold (2 μm). After that, the mold was heated to keep the tissue flat. The remaining paraffin was added to the mold to fill the gaps between holes. The TMA was made after cooling was over.

### Immunohistochemical Analysis

After deparaffinization, the activity of endogenous peroxidase was blocked on slides by using 3% H_2_O_2_ in methanol, then pretreated in sodium citrate buffer, and heated in a microwave oven (except 4-HNE), incubated with serum in BSA. Subsequently, TMA sections were incubated with corresponding primary and secondary antibody, and the reaction products were visualized with DAB. Hematoxylin was used for the counterstain of all slides. As a negative control, 1% bovine serum albumin (BSA) was used to replace the primary antibody on sections that were proven to be positive for 4-HNE, p-AMPK, Erk1/2, p-Erk1/2, CARM1, p62, LAMP1, and TFEB in the present experiments.

### Multispectral Imaging

Two colors of DAB and hematoxylin on single-stained slides or dual-stained slides were imaged by using the Nuance Multispectral Imaging System (Cambridge Research and Instrumentation Inc., Woburn, MA), and detailed procedures can be found in our previous study (14). Images at 200× magnification were obtained at 20-nm intervals across a range of 420-720 nm, and further used to create image cubes, which were then resolved into two individual monochrome images by Nuance software (v3.0.2) for subsequent image analyses. The total signal optical density (OD) values in red, green, and blue (RGB) and mass spectroscopy (MS) images were measured.

### Multispectral Imaging Statistical Analysis

Excel (Microsoft Corporation) and Prism 5.01 (GraphPad Software) were used to process the data, before-after graphs and bar charts were then obtained, and the statistical analyses were further performed. Paired *t*-test was performed for each dependent variable. According to the degrees of tumor differentiation in patients (well-differentiated group vs. moderately and poorly differentiated group), we analyzed each protein's expression in the ratio of “cancerous region/non-cancerous region” in two groups. An unpaired *t*-test was conducted for each dependent variable. ^*^p < 0.05, ^**^p < 0.01, and ^***^p < 0.001 were considered statistically significant; very significant; and extremely significant, respectively, and *p* > 0.05 was considered not significant.

## Result

### 4-HNE, Which Could Inhibit the Activity of AMPK, Was Downregulated in Cancerous Regions

**Figure 1:**
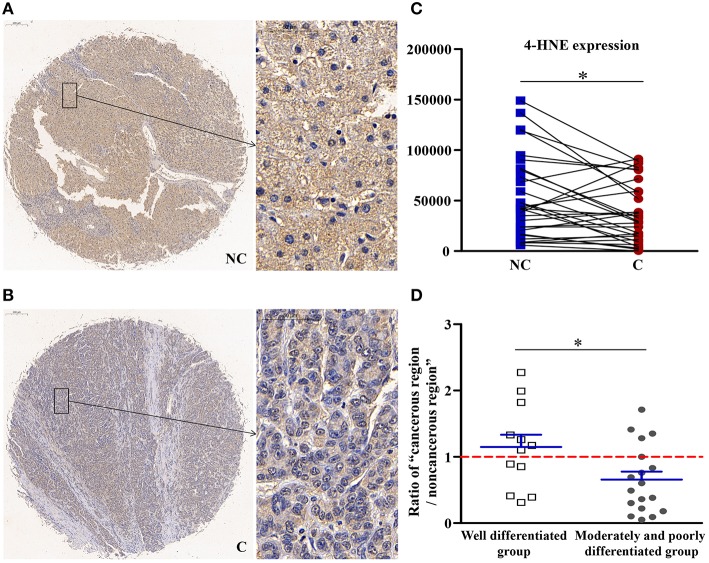
The detailed expression of 4-HNE. Representative images of 4-HNE protein expression in liver cancer's corresponding **(A)** non-cancerous (NC) and **(B)** cancerous (C) regions. **(C)** Quantification of 4-HNE expression level using multispectral image analysis in the liver cancer samples. **(D)** The ratio of “cancerous region/non-cancerous region” of 4-HNE expression in two groups (well-differentiated group vs. moderately and poorly differentiated group). The red line indicates that the ratio is equal to 1. Scale bar: 200 or 50 μm. *Denotes significant differences from the noncancerous region, or well-differentiated group with *P* < 0.05.

As shown in Figure 1, compared with non-cancerous regions, the expression of 4-HNE, an oxidative stress product, was significantly lower (*p* < 0.05) in cancerous regions. Compared with well-differentiated patients, the expression of 4-HNE in patients with moderately and poorly differentiated tumors was more significantly downregulated (*p* < 0.05). That is, as the degree of differentiation decreased, the extent of downregulation in the expression of 4-HNE increased.

### p-AMPK Upregulated in Cancerous Regions

**Figure 2:**
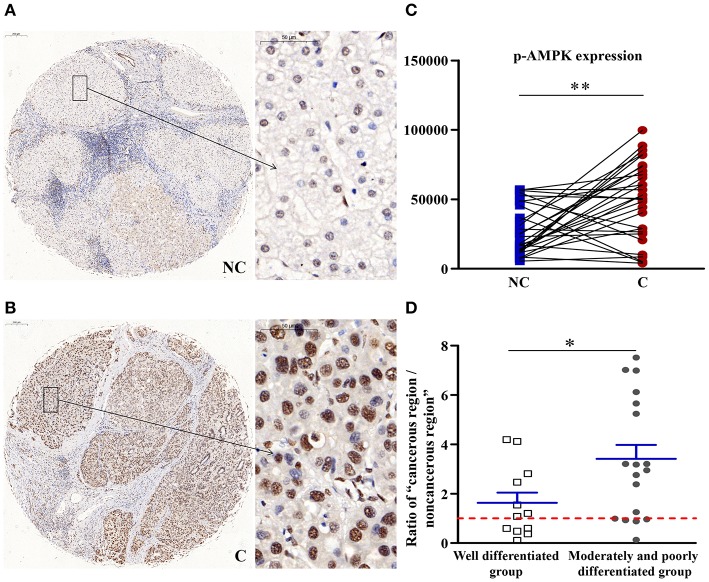
The detailed expression of p-AMPK. Representative images of p-AMPK protein expression in liver cancer's corresponding **(A)** non-cancerous (NC) and **(B)** cancerous (C) regions. **(C)** Quantification of p-AMPK expression level using multispectral image analysis in the liver cancer samples. **(D)** The ratio of “cancerous region/non-cancerous region” of p-AMPK expression in two groups (well-differentiated group vs. moderately and poorly differentiated group). The red line indicates that the ratio is equal to 1. Scale bar: 200 or 50 μm. *Denotes significant differences from the well-differentiated group with *P* < 0.05; **Denotes significant differences from the noncancerous region with *P* < 0.01.

As shown in Figure 2, compared with the non-cancerous region, the phosphorylation of the energy regulator AMPK (p-AMPK) was significantly higher in the cancerous tissues (*p* < 0.01). Compared with well-differentiated patients, the expression of p-AMPKα in patients with moderately and poorly differentiated tumors was upregulated more significantly (*p* < 0.05). As the degree of differentiation decreased, the extent of upregulation in the expression of p-AMPKα increased.

### Upregulation of Erk1/2 in Cancerous Regions

**Figure 3:**
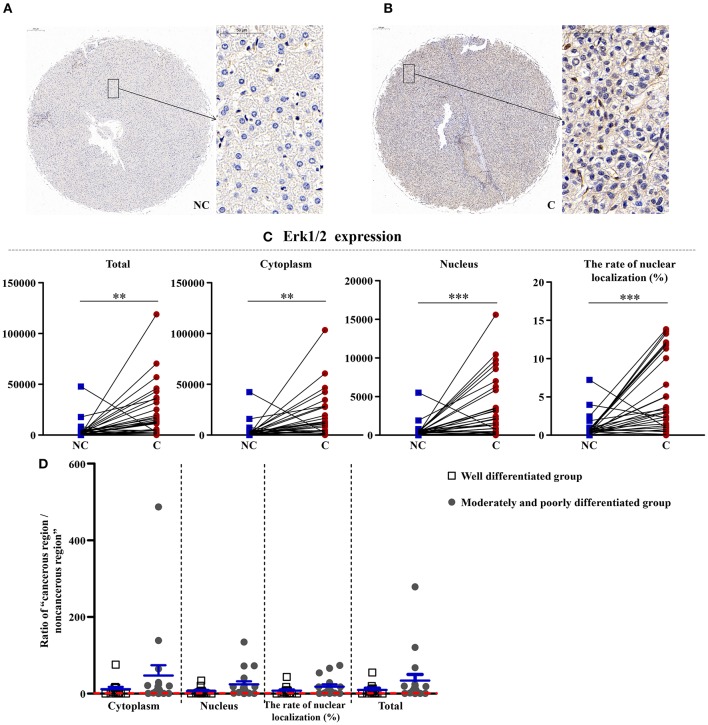
The detailed expression of Erk1/2. Representative images of Erk1/2 protein expression in liver cancer's corresponding **(A)** non-cancerous (NC) and **(B)** cancerous (C) regions. **(C)** Quantification of Erk1/2 expression (total, cytoplasmic, and nuclear expressions and the rate of nuclear localization) levels using multispectral image analysis in the liver cancer samples. **(D)** The ratio of “cancerous region/non-cancerous region” of Erk1/2 expression in two groups (well-differentiated group vs. moderately and poorly differentiated group). The red line indicates that the ratio is equal to 1. Scale bar: 200 or 50 μm. **, ***Denote significant differences from the well-differentiated group with *P* < 0.01, 0.001, respectively.

**Figure 4:**
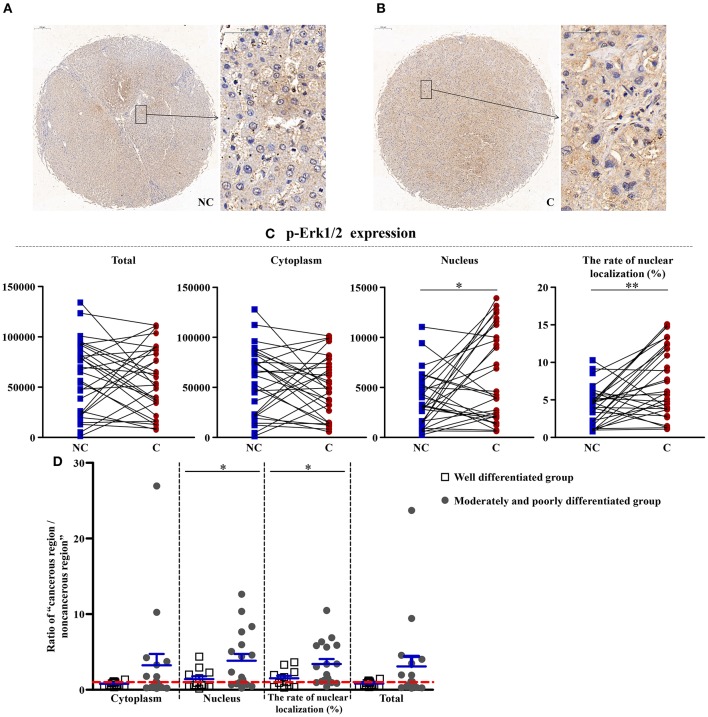
The detailed expression of p-Erk1/2. Representative images of p-Erk1/2 protein expression in liver cancer's corresponding **(A)** non-cancerous (NC) and **(B)** cancerous (C) regions. **(C)** Quantification of p-Erk1/2 expression (total, cytoplasmic, and nuclear expressions and the rate of nuclear localization) levels using multispectral image analysis in the liver cancer samples. **(D)** The ratio of “cancerous region/non-cancerous region” of p-Erk1/2 expression in two groups (well-differentiated group vs. moderately and poorly differentiated group). The red line indicates that the ratio is equal to 1. Scale bar: 200 or 50 μm. *, **Denote significant differences from the noncancerous region, or well-differentiated group with *P* < 0.05, 0.01, respectively.

As shown in Figures 3, 4, the total (*p* < 0.01), cytoplasmic (*p* < 0.01), and nuclear expressions (*p* < 0.001) and the rate of nuclear localization (*p* < 0.001) of Erk1/2 were significantly higher in the cancerous regions than that in non-cancerous regions. No significant differences in the total and cytoplasmic expressions of the p-Erk1/2 were found between the cancerous and non-cancerous regions. However, its nuclear expression (*p* < 0.05) and the rate of nuclear localization (*p* < 0.01) were significantly upregulated in the cancerous regions compared to that in non-cancerous regions. The up-regulation of Erk1/2 may be caused by the increase of p-Erk1/2 in the nucleus. Although no significant difference was occurred in the expressions of Erk1/2 in patients between the two groups, the nuclear expression (*p* < 0.05) and rate of nuclear localization (*p* < 0.05) of p-Erk1/2 showed a significant difference in the two groups. That is, as the degree of differentiation became decreased, the extent of upregulation in the nuclear expression of p-Erk1/2 increased.

### CARM1, a Downstream of AMPK, Was Higher in Cancerous Regions

**Figure 5:**
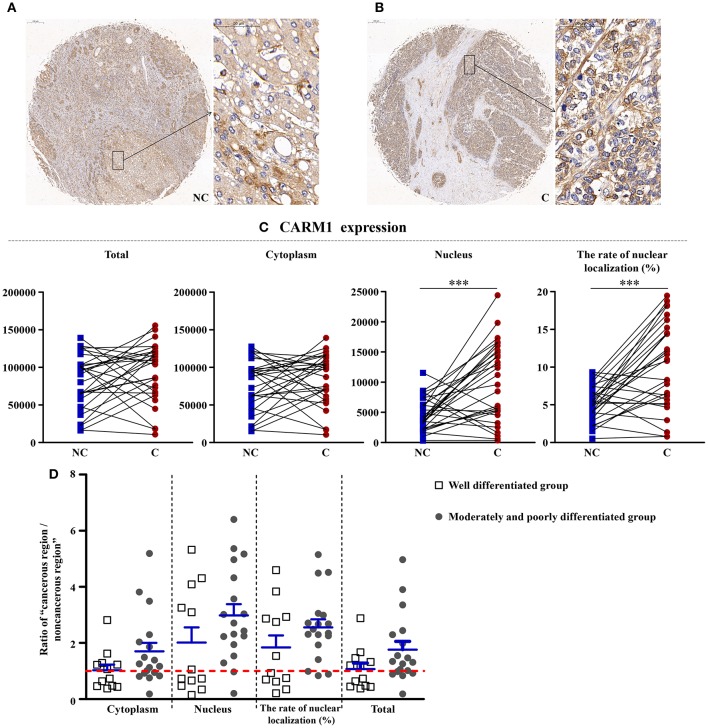
The detailed expression of CARM1. Representative images of CARM1 protein expression in liver cancer's corresponding **(A)** non-cancerous (NC) and **(B)** cancerous (C) regions. **(C)** Quantification of CARM1 expression (total, cytoplasmic, and nuclear expressions and the rate of nuclear localization) levels using multispectral image analysis in the liver cancer samples. **(D)** The ratio of “cancerous region/non-cancerous region” of CARM1 expression in two groups (well-differentiated group vs. moderately and poorly differentiated group). The red line indicates that the ratio is equal to 1. Scale bar: 200 or 50 μm. ***Denotes significant differences from the well-differentiated group with *P* < 0.001.

As shown in Figure 5, CARM1 is the downstream factor of AMPK, although the total and cytoplasm expressions of it have no significant difference, the nucleus expression (*p* < 0.001) and the rate of nucleus localization (*p* < 0.001) of CARM1 were significantly higher in cancerous region than that in non-cancerous region.

### Reduction of TFEB in Cancerous Regions

**Figure 6:**
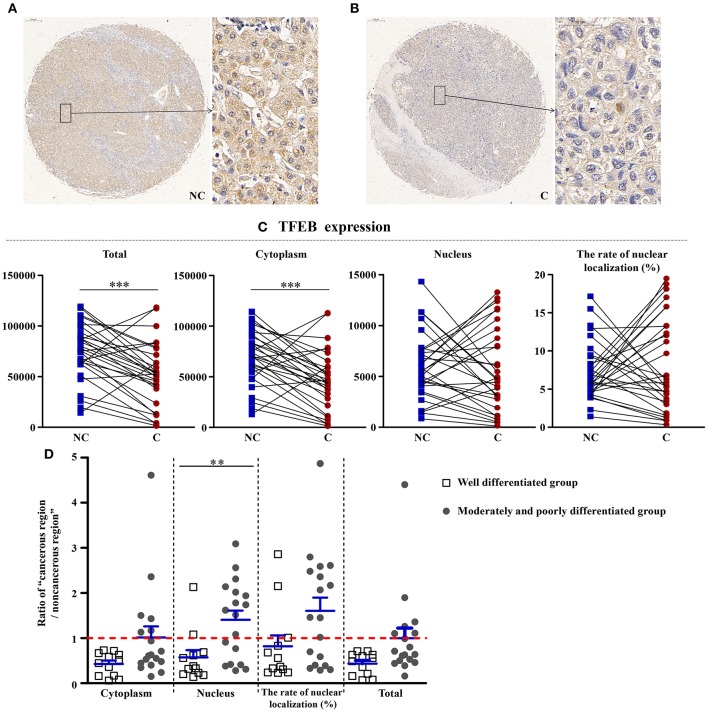
The detailed expression of TFEB. Representative images of TFEB protein expression in liver cancer's corresponding **(A)** non-cancerous (NC) and **(B)** cancerous (C) regions. **(C)** Quantification of TFEB expression (total, cytoplasmic, and nuclear expressions and the rate of nuclear localization) levels using multispectral image analysis in the liver cancer samples. **(D)** The ratio of “cancerous region/non-cancerous region” of TFEB expression in two groups (well-differentiated group vs. moderately and poorly differentiated group). The red line indicates that the ratio is equal to 1. Scale bar: 200 or 50 μm. **Denotes significant differences from the well-differentiated group with *P* < 0.01; ***Denotes significant differences from the noncancerous region with *P* < 0.001.

As shown in Figure 6, compared with non-cancerous regions, the total (*p* < 0.001) and cytoplasmic expressions (*p* < 0.001) of TFEB, a main gene that controls the biosynthesis of lysosomes, were significantly reduced in cancerous regions. However, the nuclear expression and the rate of nuclear localization had no significant difference in two regions, showing that the synthesis of lysosomes was affected in the course of liver cancer. Interestingly, compared with well-differentiated liver cancer patients, the nuclear expression of TFEB in patients with moderately and poorly differentiated cancer was upregulated more significantly (*p* < 0.01).

### The Number of Lysosomes Was Increased in Cancerous Regions

**Figure 7:**
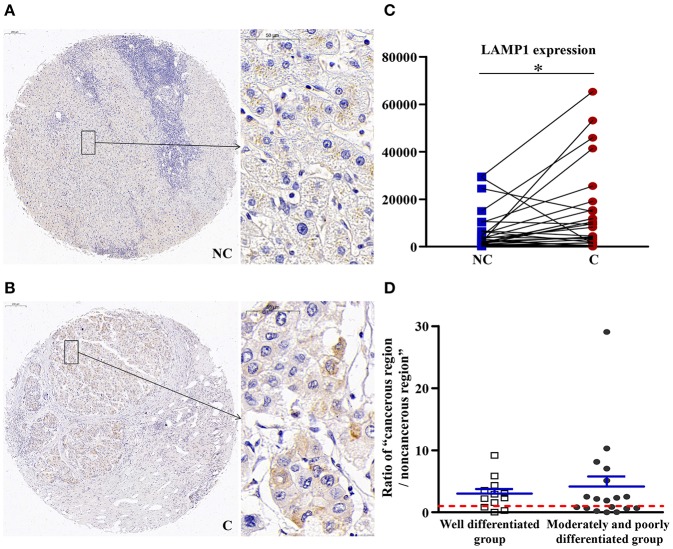
The detailed expression LAMP1. Representative images of LAMP1 protein expression in liver cancer's corresponding **(A)** non-cancerous (NC) and **(B)** cancerous (C) regions. **(C)** Quantification of LAMP1 expression level using multispectral image analysis in the liver cancer samples. **(D)** The ratio of “cancerous region/non-cancerous region” of LAMP1 expression in two groups (well-differentiated group vs. moderately and poorly differentiated group). The red line indicates that the ratio is equal to 1. Scale bar: 200 or 50 μm. *Denotes significant differences from the noncancerous region with *P* < 0.05.

As shown in Figure 7, compared with non-cancerous regions, the expression of LAMP1 was significantly higher in cancerous regions (*p* < 0.05), which suggested an increase in the number of lysosomes in cancerous regions.

### Autophagy in Cancerous Regions Was More Active Than in Non-cancerous Regions

**Figure 8:**
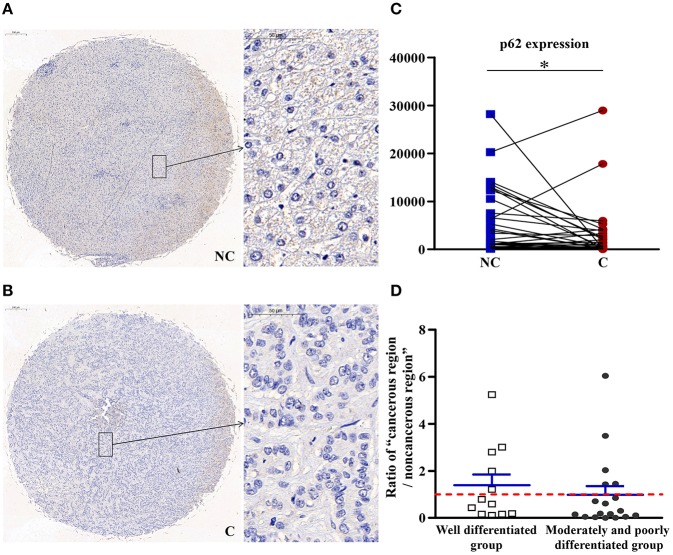
The detailed expression of p62. Representative images of p62 protein expression in liver cancer's corresponding **(A)** non-cancerous (NC) and **(B)** cancerous (C) regions. **(C)** Quantification of p62 expression level using multispectral image analysis in the liver cancer samples. **(D)** The ratio of “cancerous region/non-cancerous region” of p62 expression in two groups (well-differentiated group vs. moderately and poorly differentiated group). The red line indicates that the ratio is equal to 1. Scale bar: 200 or 50 μm. *Denotes significant differences from the noncancerous region with *P* < 0.05.

As shown in Figure 8, compared with non-cancerous regions, p62/SQSTM1, a substrate specifically degraded in autophagy, was negatively correlated with autophagy and significantly downregulated (*p* < 0.05) in cancerous regions, suggesting that autophagy in cancerous regions was more active than in non-cancerous regions.

## Discussion

Liver cancer is the sixth most commonly diagnosed cancer worldwide, resulting in hundreds of thousands of deaths every year. The Global Cancer Observatory (GCO) estimated that it would have 1.36 million (+61.9%) incident cases and 1.28 million (+64.3%) deaths of liver cancer from 2018 to 2040 worldwide^2^. Recently, more and more researches have focused on the involvement of autophagy in liver cancer. Researchers believe that clarifying the molecular mechanisms underlying the autophagy modulation and liver cancer development can promote the translational studies, and new therapeutic strategies will be ultimately developed for liver cancer.

### An Oxidative Product, 4-HNE, Was Downregulated in Cancerous Regions

Autophagy is essential for clearing nonfunctional organelles and proteins during the process of oxidative stress, and in this role, it serves an essential antioxidant function (2). Electrophiles, such as 4-HNE can lead to the dysregulation of cell signaling, which plays a main role in the pathogenesis of chronic inflammatory liver disease. It has also been effective in decreasing the cellular proliferation in several cell types, such as breast cancer cells and prostate (15, 16), and it is involved in the pathogenesis of several degenerative diseases and cancer types (17). Some studies (18, 19) have shown that 4-HNE-induced aggregates can inhibit autophagy. However, the mechanism underlying 4-HNE regulation of autophagy remains unknown. Recently, Zhong et al. (20) found that 4-HNE-protein adducts in tumor tissues of hepatocellular carcinoma decreased gradually. In our study, 4-HNE was significantly downregulated in cancerous regions, which suggests that its inhibitory effect on the AMPK had diminished. Additionally, compared with well-differentiated patients, the expression of 4-HNE in patients with moderately and poorly differentiated tumors was more significantly downregulated. That is, as the degree of differentiation decreased, the extent of downregulation in the expression of 4-HNE increased, which suggests that 4-HNE may be an indicator of the degree of differentiation of liver cancer.

### When AMPK, a Direct Target of 4-HNE, Was Upregulated in Cancerous Tissues, Energy Consumption Increased

In response to environmental stress or nutritional factors, which will deplete intracellular adenosine triphosphate (ATP), AMPK is activated by the allosteric binding of AMP and the phosphorylation via upstream AMPK kinases (21). Activation of AMPK plays an important role in maintaining homeostasis of cellular energy under stress conditions at both cellular and physiological level (22–24). An anti-cancer function after the activation of AMPK has been proposed (25, 26). Activation of AMPK upregulates the autophagic pathway. We found that the expression of p-AMPK in cancerous regions was much more higher than that in non-cancerous regions, which means that autophagy is upregulated in liver cancer. Compared with well-differentiated patients, the expression of p-AMPKα in patients with moderately and poorly differentiated tumors was upregulated more significantly. As the degree of differentiation decreased, the extent of upregulation in the expression of p-AMPKα increased. 4-HNE can directly action on AMPK. In HepG2 cells, 4-HNE works in two ways. Firstly, 4-NHE can inhibit the activation of AMPK by H_2_O_2_. Secondly, 4-NHE inhibits the activity of AMPK via the direct modification of recombinant AMPK (27). Our results showed that the 4-HNE'capacity of inhibiting the activity of AMPK decreased, the expression of p-AMPK was upregulated, and the energy consumption of tumor cells increased, which affected subsequent changes.

### The Downstream of AMPK, Nuclear Expression of Erk1/2 and p-Erk1/2 U-Regulated in Cancerous Tissues, and p-Erk1/2 Is Associated With Liver Differentiation

Aberrant activation of the ERK pathway has been confirmed to be an essential feature for various human tumors (9). The activated Erk1/2 can phosphorylate various downstream substrates involved in the cellular responses of cell survival, cell motility, cell proliferation, and cell differentiation. Recently, Xiao et al. (11) reported that Erk1/2 can promote the ATG7-dependent autophagy, which is a novel beneficial effect for Erk1/2 in liver steatosis. The MEK/ERK pathway was upstream of TSC and mTOR and downstream of AMPK (8). The MEK/ERK module can regulate the Beclin 1 level via the AMPK-MEK/ERK-TSC-mTOR pathway, thus regulating the autophagy. In our study, the total, cytoplasmic, and nuclear expressions and the rate of nuclear localization of Erk1/2 were significantly higher in the cancerous regions than that in non-cancerous regions, which was in accordance with other scientists' studies (28). Total and cytoplasmic expressions of p-Erk1/2 showed no significant difference between the cancerous and non-cancerous regions. However, its nuclear expression and the rate of nuclear localization were significantly upregulated in the cancerous regions compared to that in non-cancerous regions. The upregulation of Erk1/2 may be caused by the increase of p-Erk1/2 in the nucleus. Although no significant difference was occurred in the expressions of Erk1/2 in patients between the two groups, the nuclear expression and rate of nuclear localization of p-Erk1/2 showed significant difference in the two groups. That is, as the degree of differentiation decreased, the extent of upregulation in the nuclear expression of p-Erk1/2 increased. Combined with the downregulation of p-AMPK, this further confirmed the up-regulation of autophagy in cancerous regions.

### Another Downstream of AMPK, Nuclear Expression of CARM1, Increased in Cancerous Tissues

Biochemically, as a cofactor for several transcription factors and nuclear hormone receptors, the histone methyltransferase of CARM1 belongs to a family of arginine methyltransferases, and is involved in the tumorigenesis of various cancers, such as ovarian cancer (29). Osada et al. (30) found that the expression of CARM1 during hepatocellular carcinogenesis increased in adenomas and was aberrant in carcinomas. Shin et al. (13) recently found that CARM1 was a key component among the AMPK-SKP2-CARM1 signaling axis in autophagy in mammals. In the current study, the nucleus expression and the rate of nucleus localization of CARM1 were significantly higher in cancerous regions than that in non-cancerous regions, which is in accordance with the results mentioned above. Combined with the downregulation of p-AMPK, this further confirmed the upregulation of autophagy in cancerous regions.

### Reduction of Total Expression of TFEB in Cancerous Tissues, Indicating Impaired Lysosomal Synthesis

Autophagy has been found to selectively degrade specific cellular constituents in the lysosome (31). TFEB controls the process of lysosomal biogenesis and autophagy. It always localizes to the cytosol, and will mobilize into the nucleus when lysosomal function is compromised or starvation conditions was reached (32). Study has shown that TFEB is regulated by Erk1/2 (33). TFEB overexpression can lead to an increase of autophagosomes and autophagic flux, generation of new lysosomes, and clearance of storage material in several disordered lysosomal storage through the promotion of lysosomal secretion (34). Overexpression of TFEB was occurred for the patients with renal cell carcinoma (35), and it was confirmed to accelerate tumorigenesis by inducing various oncogenic signals. Recently, a study (36) showed that TFEB in the liver of mice reduced the liver damage caused by alcohol. In our study, compared with noncancerous regions, the total and cytoplasmic expressions of TFEB were significantly reduced in cancerous regions. However, the nuclear expression and the rate of nuclear localization had no significant difference in two regions, showing that the synthesis of lysosomes was affected in the course of liver cancer. Interestingly, compared with well-differentiated liver cancer patients, the nuclear expression of TFEB in patients with moderately and poorly differentiated tumors was upregulated significantly.

### The Downstream of TFEB, Expression of LAMP1 Was Upregulated in Cancerous Tissues, Indicating an Increase in the Number of Lysosomes in Cancerous Tissues

LAMP1, as a downstream effector of TFEB (37), is a key protein in the biogenesis of lysosomal. It has assumed importance as a marker of lysosomes in various biological studies, with the most abundant lysosomal membrane proteins being LAMP-1, which represents membrane proteins of the lysosomal membrane. The expression of LAMP-1 occurred on the surface of various cancer cells, such as colon cancer (38). LAMP1 can significantly promote the development, proliferation, and metastasis of cancer, and the downregulation of LAMP1 can inhibit the metastasis of cancer (39). In our study, LAMP1 was upregulated significantly in cancerous regions, which suggested an increase in the number of lysosomes in cancerous regions. Combined with the results of TFEB mentioned above, we hypothesized that the function of lysosomes was not seriously damaged in the well-differentiated stage of liver cancer. In moderately and poorly differentiated tumors, the damage to the lysosomes was aggravated. The nuclear expression of TFEB in cancerous tissues was significantly increased, which led to upregulation of LAMP1 expression, but the factors that affect changes in LAMP1 in well-differentiated tumors require further research.

### Specific Autophagy Degradation Substrate p62/SQSTM1 Was Downregulated in Cancerous Tissues, Indicating That Autophagy May Be More Active in Cancerous Areas

The expression of many autophagy-lysosomal genes, including p62/SQSTM1, was controlled by TFEB (40). p62 can achieve their degradation in the lysosome by linking ubiquitinated proteins to the autophagic machinery (41). The amount of p62/SQSTM1 will markedly accumulates when autophagy is suppressed, while decreases when autophagy is activated. Therefore, p62 is a common marker for autophagic flux (42). The expression of p62 was downregulated in cancerous regions, which suggested that autophagy in cancerous regions was more active than in non-cancerous regions.

## Conclusion

Based on these facts, we hypothesized that, during the progression of liver tumors, there were AMPK-ERK/CARM1 autophagic signaling pathways. Followed by oxidative stress, the expression of 4-HNE was downregulated, and hence its ability to inhibit AMPK decreased. This subsequently led to increase in phosphorylation and alteration in the nuclear localization of AMPK, which further enhanced the rate of nuclear localization of p-Erk1/2 and elevated the expression of Erk1/2. In addition, the rate of nuclear localization of CARM1, another downstream of AMPK, was also enlarged. Other changes, including the reduction of TFEB and p62 and the up-regulation of LAMP1, were also observed. The above-mentioned changes in signals indicated an upregulation of autophagy in cancerous regions, which means that overactivated autophagy plays an important role in liver cancer.

## Data Availability Statement

All datasets generated for this study are included in the article/supplementary material.

## Ethics Statement

The studies involving human participants were reviewed and approved by the Committee on the Ethics of Experiments of the South-Central University for Nationalities in China (Permit Number: 2017-SCUEC-MEC-007). The patients/participants provided their written informed consent to participate in this study.

## Author Contributions

Q-FQ, Y-SL, and H-BT performed the experiments. Q-FQ, X-JL, Y-SL, WZ, G-HT, H-CS, and H-BT analyzed the data. Y-SL, WZ, G-HT, H-CS, and H-BT contributed reagents, materials, and analysis tools. Q-FQ, Y-SL, WZ, G-HT, H-CS, and H-BT wrote the paper.

### Conflict of Interest

The authors declare that the research was conducted in the absence of any commercial or financial relationships that could be construed as a potential conflict of interest.

## Footnotes

^1^https://www.who.int/news-room/fact-sheets/detail/cancer

^2^http://gco.iarc.fr/tomorrow/home

## References

[1] Choi AM Ryter SW Levine B Autophagy in human health and disease. N Engl J Med. (2013) 368:1845–6. 10.1056/NEJMc1303158 23656658

[2] Sarkar S Ravikumar B Floto RA Rubinsztein DC. Rapamycin and mTOR-independent autophagy inducers ameliorate toxicity of polyglutamine-expanded huntingtin and related proteinopathies. Cell Death Differ. (2009) 16:46–56. 10.1038/cdd.2008.110 18636076

[3] Dodson M Liang Q Johnson MS Redmann M Fineberg N Darley-Usmar VM. Inhibition of glycolysis attenuates 4-hydroxynonenal-dependent autophagy and exacerbates apoptosis in differentiated SH-SY5Y neuroblastoma cells. Autophagy. (2013) 9: 1996–2008. 10.4161/auto.26094 24145463 PMC4028343

[4] Chaudhary P Sharma R Sharma A Vatsyayan R Yadav S Singhal SS. Mechanisms of 4-hydroxy-2-nonenal induced pro- and anti-apoptotic signaling. Biochemistry. (2010) 49:6263–75. 10.1021/bi100517x 20565132 PMC2957295

[5] Zhang Z Wang S Zhou S Yan X Wang Y Chen J. Sulforaphane prevents the development of cardiomyopathy in type 2 diabetic mice probably by reversing oxidative stress-induced inhibition of LKB1/AMPK pathway. J Mol Cell Cardiol. (2014) 77:42–52. 10.1016/j.yjmcc.2014.09.022 25268649

[6] Hardie DG. AMP-activated/SNF1 protein kinases: conserved guardians of cellular energy. Nat Rev Mol Cell Biol. (2007) 8:774–85. 10.1038/nrm2249 17712357

[7] Kim J Kundu M Viollet B Guan KL. AMPK and mTOR regulate autophagy through direct phosphorylation of Ulk1. Nat Cell Biol. (2011) 13:132–41. 10.1038/ncb2152 21258367 PMC3987946

[8] Wang J Whiteman MW Lian H Wang G Singh A Huang D. A non-canonical MEK/ERK signaling pathway regulates autophagy via regulating Beclin 1. J Biol Chem. (2009) 284:21412–24. 10.1074/jbc.M109.026013 19520853 PMC2755866

[9] Kohno M Pouyssegur J. Targeting the ERK signaling pathway in cancer therapy. Ann Med. (2006) 38:200–11. 10.1080/07853890600551037 16720434

[10] Thapalia BA Zhou Z Lin X. Sauchinone augments cardiomyocyte viability by enhancing autophagy proteins -PI3K, ERK(1/2), AMPK and Beclin-1 during early ischemia-reperfusion injury *in vitro*. Am J Transl Res. (2016) 8:3251–65. 27508047 PMC4969463

[11] Xiao Y Liu H Yu J Zhao Z Xiao F Xia T. Activation of ERK1/2 ameliorates liver steatosis in leptin receptor-deficient (db/db) mice via stimulating ATG7-dependent autophagy. Diabetes. (2016) 65:393–405. 10.2337/db15-1024 26581593

[12] Yin Y Zhao Y Han S Zhang N Chen H Wang X Autophagy-ERK1/2-involved disinhibition of hippocampal neurons contributes to the pre-synaptic toxicity induced by a β42 exposure. J Alzheimers Dis. (2017) 59: 851–69. 10.3233/JAD-170246 28697568

[13] Shin HJ Kim H Oh S Lee JG Kee M Ko HJ. AMPK-SKP2-CARM1 signalling cascade in transcriptional regulation of autophagy. Nature. (2016) 534:553–7. 10.1038/nature18014 27309807 PMC5568428

[14] Li YS Wang JX Jia MM Liu M Li XJ Tang HB. Dragon's blood inhibits chronic inflammatory and neuropathic pain responses by blocking the synthesis and release of substance P in rats. J Pharmacol Sci. (2012) 118:43–54. 10.1254/jphs.11160FP 22198006

[15] Covey TM Edes K Coombs GS Virshup DM Fitzpatrick FA. Alkylation of the tumor suppressor PTEN activates Akt and β-catenin signaling: a mechanism linking inflammation and oxidative stress with cancer. PLoS ONE. (2010) 5:e13545. 10.1371/journal.pone.0013545 20975834 PMC2958828

[16] Pettazzoni P Pizzimenti S Toaldo C Sotomayor P Tagliavacca L Liu S. Induction of cell cycle arrest and DNA damage by the HDAC inhibitor panobinostat (LBH589) and the lipid peroxidation end product 4-hydroxynonenal in prostate cancer cells. Free Radic Biol Med. (2011) 50:313–22. 10.1016/j.freeradbiomed.2010.11.011 21078383

[17] Uchida K. 4-Hydroxy-2-nonenal: a product and mediator of oxidative stress. Prog Lipid Res. (2003) 42:318–43. 10.1016/S0163-7827(03)00014-6 12689622

[18] Dodson M Wani WY Redmann M Benavides GA Johnson MS Ouyang X. Regulation of autophagy, mitochondrial dynamics, and cellular bioenergetics by 4-hydroxynonenal in primary neurons. Autophagy. (2017) 13:1828–40. 10.1080/15548627.2017.1356948 28837411 PMC5788494

[19] Bonet-Ponce L Saez-Atienzar S da Casa C Sancho-Pelluz J Barcia JM Martinez-Gil N. Rotenone induces the formation of 4-hydroxynonenal aggresomes. Role of ROS-mediated tubulin hyperacetylation and autophagic flux disruption. Mol Neurobiol. (2016) 53:6194–208. 10.1007/s12035-015-9509-3 26558631

[20] Zhong H Xiao M Zarkovic K Zhu M Sa R Lu J. Mitochondrial control of apoptosis through modulation of cardiolipin oxidation in hepatocellular carcinoma: a novel link between oxidative stress and cancer. Free Radic Biol Med. (2017) 102:67–76. 10.1016/j.freeradbiomed.2016.10.494 27838437

[21] Jung SN Park IJ Kim MJ Kang I Choe W Kim SS. Down-regulation of AMP-activated protein kinase sensitizes DU145 carcinoma to Fas-induced apoptosis via c-FLIP degradation. Exp Cell Res. (2009) 315:2433–41. 10.1016/j.yexcr.2009.05.018 19477172

[22] Shackelford DB Shaw RJ. The LKB1-AMPK pathway: metabolism and growth control in tumour suppression. Nat Rev Cancer. (2009) 9:563–75. 10.1038/nrc2676 19629071 PMC2756045

[23] Mihaylova MM Shaw RJ. The AMPK signalling pathway coordinates cell growth, autophagy and metabolism. Nat Cell Biol. (2011) 13:1016–23. 10.1038/ncb2329 21892142 PMC3249400

[24] Vakana E Altman JK Platanias LC. Targeting AMPK in the treatment of malignancies. J Cell Biochem. (2012) 113:404–9. 10.1002/jcb.23369 21928327

[25] Zhao Z Feng L Wang J Cheng D Liu M Ling M. NPC-26 kills human colorectal cancer cells via activating AMPK signaling. Oncotarget. (2017) 8 18312–21. 10.18632/oncotarget.15436 28407688 PMC5392330

[26] Law BY Mok SW Chan WK Xu SW Wu AG Yao XJ. Hernandezine, a novel AMPK activator induces autophagic cell death in drug-resistant cancers. Oncotarget. (2016) 7:8090–104. 10.18632/oncotarget.6980 26811496 PMC4884978

[27] Shearn CT Backos DS Orlicky DJ Smathers-McCullough RL Petersen DR. Identification of 5' AMP-activated kinase as a target of reactive aldehydes during chronic ingestion of high concentrations of ethanol. J Biol Chem. (2014) 289:15449–62. 10.1074/jbc.M113.543942 24722988 PMC4140901

[28] Zhang X Yang H Yue S He G Qu S Zhang Z. The mTOR inhibition in concurrence with ERK1/2 activation is involved in excessive autophagy induced by glycyrrhizin in hepatocellular carcinoma. Cancer Med. (2017) 6:1941–51. 10.1002/cam4.1127 28675698 PMC5548872

[29] Nakayama N Sakashita G Nariai Y Kato H Sinmyozu K Nakayama JI. Cancer-related transcription regulator protein NAC1 forms a protein complex with CARM1 for ovarian cancer progression. Oncotarget. (2018) 9:28408–20. 10.18632/oncotarget.25400 29983869 PMC6033357

[30] Osada S Suzuki S Yoshimi C Matsumoto M Shirai T Takahashi S. Elevated expression of coactivator-associated arginine methyltransferase 1 is associated with early hepatocarcinogenesis. Oncol Rep. (2013) 30:1669–74. 10.3892/or.2013.2651 23912631

[31] Chourasia AH Boland ML Macleod KF. Mitophagy and cancer. Cancer Metab. (2015) 3:4. 10.1186/s40170-015-0130-8 25810907 PMC4373087

[32] Sardiello M Palmieri M di Ronza A Medina DL Valenza M Gennarino VA. A gene network regulating lysosomal biogenesis and function. Science. (2009) 325:473–7. 10.1126/science.1174447 19556463

[33] Shen HM Mizushima N. At the end of the autophagic road: an emerging understanding of lysosomal functions in autophagy. Trends Biochem Sci. (2014) 39:61–71. 10.1016/j.tibs.2013.12.001 24369758

[34] Medina DL Fraldi A Bouche V Annunziata F Mansueto G Spampanato C. Transcriptional activation of lysosomal exocytosis promotes cellular clearance. Dev Cell. (2011) 21:421–30. 10.1016/j.devcel.2011.07.016 21889421 PMC3173716

[35] Calcagni A Kors L Verschuren E De Cegli R Zampelli N Nusco E. Modelling TFE renal cell carcinoma in mice reveals a critical role of WNT signaling. Elife. (2016) 5:e17047. 10.7554/eLife.17047 27668431 PMC5036965

[36] Chao XJ Wang SG Zhao K Li Y Williams JA Li TG. Impaired TFEB-mediated lysosome biogenesis and autophagy promote chronic ethanol-induced liver injury and steatosis in mice. Gastroenterology. (2018) 155:865–6. 10.1053/j.gastro.2018.05.027 29782848 PMC6120772

[37] Li F Lang F Zhang H Xu L Wang Y Hao E. Role of TFEB Mediated autophagy, oxidative stress, inflammation, and cell death in endotoxin induced myocardial toxicity of young and aged mice. Oxid Med Cell Longev. (2016) 2016:5380319. 10.1155/2016/5380319 27200146 PMC4856916

[38] Piao S Amaravadi RK. Targeting the lysosome in cancer. Ann N Y Acad Sci. (2016) 1371:45–54. 10.1111/nyas.12953 26599426 PMC4879098

[39] Agarwal AK Srinivasan N Godbole R More SK Budnar S Gude RP. Role of tumor cell surface lysosome-associated membrane protein-1 (LAMP1) and its associated carbohydrates in lung metastasis. J Cancer Res Clin Oncol. (2015) 141:1563–74. 10.1007/s00432-015-1917-2 25614122 PMC11823972

[40] Palmieri M Impey S Kang H di Ronza A Pelz C Sardiello M. Characterization of the CLEAR network reveals an integrated control of cellular clearance pathways. Hum Mol Genet. (2011) 20:3852–66. 10.1093/hmg/ddr306 21752829

[41] Pankiv S Clausen TH Lamark T Brech A Bruun JA Outzen H. p62/SQSTM1 binds directly to Atg8/LC3 to facilitate degradation of ubiquitinated protein aggregates by autophagy. J Biol Chem. (2007) 282:24131–45. 10.1074/jbc.M702824200 17580304

[42] Cheng Y Ren X Hait WN Yang JM. Therapeutic targeting of autophagy in disease: biology and pharmacology. Pharmacol Rev. (2013) 65:1162–97. 10.1124/pr.112.007120 23943849 PMC3799234

